# Anti-inflammatory and uric acid lowering effects of Euodiae fructus on hyperuricemia and gout mice

**DOI:** 10.3389/fphar.2024.1296075

**Published:** 2024-04-19

**Authors:** Zhilei Wang, Jingwen Liu, Yu Mou, Wenhao Liao, Yuchen Li, Juan Liu, Jianyuan Tang

**Affiliations:** ^1^ TCM Regulating Metabolic Diseases Key Laboratory of Sichuan Province, Hospital of Chengdu University of Traditional Chinese Medicine, Chengdu, China; ^2^ Hospital of Chengdu University of Traditional Chinese Medicine, Chengdu, China

**Keywords:** hyperuricemia, gout, uric acid, Euodiae fructus, NLRP3 inflammasome

## Abstract

The metabolic disease hyperuricemia (HUA) is caused by presence of excessive serum uric acid (UA), which leads to an increased risk of chronic kidney disease and gout. As a widely used traditional Chinese medicine, Euodiae fructus (ER) has strong anti-inflammatory and analgesic effects, however, its therapeutic effects on HUA and gout have not been investigated. To investigate the potential effects and underlying mechanisms, the effect of ER on proinflammatory cytokines and NLRP3 inflammasome activation was studied in mouse bone marrow macrophages. Moreover, a mouse model of HUA and gouty arthritis was established by coadministration of potassium oxonate (PO) and monosodium urate crystals to mice fed a high-fat diet (HFD) for 37 consecutive days. Oral administration of ER aqueous extract was given 1 hour later after the injection of PO for 10 days. Our study showed that ER is a powerful NLRP3 inhibitor in mouse macrophages. Most importantly, ER (0.75 g/kg) treatment substantially decreased the ankle joint thickness ratio, serum UA, creatinine and blood urea nitrogen levels (*p* < 0.05). Additionally, ER (0.75 g/kg) dramatically reversed the increases in renal urate transporter 1 (URAT1) and glucose transporter 9 (GLUT9) as well as the decreases in organic anion transporter 1 (OAT1) and ATP binding cassette subfamily G member 2 (ABCG2) levels (*p* < 0.05). Moreover, ER (0.75 g/kg) markedly ameliorated the production of the serum inflammatory cytokines IL-1β and TNF-α (*p* < 0.01), and improved the activation of NLRP3 inflammasome signaling in the kidneys. Taken together, these data indicate that ER, a powerful and specific NLRP3 inhibitor, has multiple anti-HUA, anti-gout and anti-inflammatory effects. Our investigation is designed to experimentally support the conventional use of ER-containing classical herbal formulas in the treatment of HUA-related disorders and may add a new dimension to the clinical application of ER.

## 1 Introduction

Hyperuricemia (HUA) is a common metabolic dysfunction that is most commonly attributed to the overproduction and/or inappropriate excretion of uric acid (UA). The incidence of HUA is rapidly increasing worldwide and may be associated with a variety of genetic and environmental factors, including exposure to pollutants and improper lifestyle factors (Li et al., 2020a; Danve et al., 2021; Wu et al., 2022). When serum UA accumulates in peripheral joints as monosodium urate (MSU) crystals, the immune response of the body can cause a number of gouty-related diseases, including severe gouty arthritis, gouty arthropathy, UA urolithiasis, gouty nephropathy, and nephrolithiasis (Ragab et al., 2017; Dalbeth et al., 2019). A large amount of evidence shows that gout is not only an independent risk factor for obesity, fatty liver disease, atherosclerosis, diabetes and other diseases, but also an independent predictor of premature death (Dehlin et al., 2020; Kimura et al., 2021; Copur et al., 2022; Jiang et al., 2023).

Although gout and HUA are common diseases in humans, they are still poorly treated owing to the lack of effective therapeutic options. Currently, there are several side effects, such as gastrointestinal reactions, skin allergic reactions, and liver and kidney damage, in the chinical treatment of gout, which can affect patient compliance. The xanthine oxidase inhibitor allopurinol is the first-line drug for the treatment of HUA, and the common side effects include skin mucosal damage and digestive system damage (Tsai and Yeh, 2010). Benzbromarone, a drug that promotes UA excretion, can negatively affect liver and kidney function (Sun et al., 2018; Ye et al., 2019). The therapeutic dose of colchicine is close to the toxic dose used during acute gout attack (Finkelstein et al., 2010). Non-steroidal anti-inflammatory drugs are also the drugs of choice for the treatment of gout and work primarily by inhibiting cyclooxygenase-2 activity, with common side effects including renal damage and central nervous system toxicity (Singh et al., 1994; Drożdżal et al., 2021). Therefore, alternative anti-inflammatory and urate-lowering agents are urgently needed to maximize drug efficacy and safety.

In the past few years, a variety of investigations have addressed the critical role of NLRP3 inflammasome activation in the onset and development of HUA and gout (Martinon et al., 2006; So and Martinon, 2017; Dalbeth et al., 2021). NLRP3 inflammasome can activate caspase-1, which can cleave pro-IL-1β to produce the mature cytokine IL-1β (Huang et al., 2021). Mature IL-1β leads to the recruitment of neutrophils and the release of additional inflammatory mediators, resulting in an amplification of the inflammatory cascade. In addition, the secretion of the inflammatory cytokine IL-1β, an important inflammatory mediator in acute gouty arthritis, is one of the major clinical manifestations of the early onset of gout and is the initiating factor of inflammatory signaling activation. NLRP3 inflammasome is an inflammatory sensor of metabolic disorders that mediates the development of gout, and inhibiting the NLRP3 inflammasome might be a promising therapeutic strategy for the prevention and management of gout.

The advantage of traditional Chinese medicine in the treatment of gout lies in its overall regulation. Through multiple and multitarget regulatory mechanisms, the host’s UA level can reach a dynamic equilibrium, which has clinical advantages in accordance with the characteristics of the disease. As a widely used traditional Chinese medicine, Euodiae fructus (ER), commonly known as “Wuzhuyu” in Chinese, has strong anti-inflammatory and analgesic effects (Liao et al., 2011; Zhang et al., 2020). The ER is from *Rutaceae* and is the dried, nearly ripe fruit of *Euodia rutaecarpa* (Juss.) Benth., *E. rutaecarpa* (Juss.) Benth. var. officinalis (Dode) Huang, or *E. rutaecarpa* (Juss.) Benth. var. bodinieri (Dode) Huang (Chinese Pharmacopoeia, 2020). The secondary metabolites of ER mainly include evodiamine, rutaecarpine, and evocarpine, all of which exhibit potent pharmacological effects. Evodiamine is therapeutically effective at treating ulcerative colitis, various cancers, and liver diseases (Wang et al., 2020a; Li et al., 2020b; Wang et al., 2023). Rutaecarpine plays a key role in cardiovascular protection and multiple inflammatory diseases (Tian et al., 2019; Li et al., 2023). The pharmacological action of evocarpine is characterized mainly by antimicrobial activity (Kano et al., 1991; Wube et al., 2011). However, the anti-HUA and anti-gout effects of ER and its secondary metabolites have not been elucidated.

In summary, the screening and discovery of safe and efficient traditional Chinese medicines and their active metabolites is a new direction for the development of anti-gout drugs. The ER has potent anti-inflammatory effects, and its active metabolites, evodiamine and rutaecarpine, significantly inhibit NLRP3 inflammasome activation (Shen et al., 2019; Ding et al., 2020; Luo et al., 2020). Targeting NLRP3 inflammasome, a sensor of metabolic stress, may be a key strategy for the prevention and treatment of gout. Therefore, the present study aimed to clarify the biological effect of ER on the regulation of the inflammatory microenvironment through the targeting of NLRP3 inflammasome. Moreover, the mouse model of HUA induced by high-fat diet (HFD) and potassium oxonate (PO) and the acute gouty arthritis induced by MSU crystals were established. We further explored the anti-HUA and anti-gout effects of ER in the *in vivo* model through the modulation of NLRP3 inflammasome. In addition, mouse kidney tissue metabolomics was used to establish the relationship between metabolites and physiological status and to understand the biochemical processes and biological events of ER in the prevention and treatment of HUA and gout from the metabolic perspective. This study deepens the scientific understanding of ER, provides potential drug candidates for the treatment of HUA and gout, and can help to establish quality control methods and standards for ER and related preparations.

## 2 Materials and methods

### 2.1 Mice

Six-to eight-week-old C57BL/6J mice (Chengdu Yaokang Biotechnology Co., Ltd., Chengdu, China) weighing 18–22 g were free of specific pathogens and housed at 25°C ± 5°C and 65%–70% relative humidity under a 12-h light/12-h dark cycle with unlimited access to food and water throughout the study unless otherwise needed. All animal experimental protocols were authorized by the Animal Care Committee of Chengdu University of Traditional Chinese Medicine.

### 2.2 Antibodies

An anti-mouse caspase-1 antibody (AG-20B-0042) and an anti-NLRP3 antibody (AG-20B-0014) were purchased from Adipogen. The anti-mouse IL-1β antibody (AF-401-NA) was obtained from the R&D Systems. Anti-ASC (67824) was provided by Cell Signaling Technology. The GLUT9 polyclonal antibody (26486-1-AP), ABCG2 polyclonal antibody (27286-1-AP), URAT1 polyclonal antibody (14937-1-AP), GAPDH monoclonal antibody (60004-1-1g), Lamin B1 polyclonal antibody (12987-1-AP), goat anti-mouse IgG (SA00001-1), and goat anti-rabbit IgG (SA00001-2) were obtained from Proteintech.

### 2.3 Metabolites in the ER aqueous extract

#### 2.3.1 Extraction of ER aqueous extract

The dried ER (Batch No: 231001) was obtained from Sichuan Jinlin Pharmaceutical Co., Ltd. (Emeishan, China). The voucher specimen of ER is deposited in the laboratory of TCM Regulating Metabolic Diseases Key Laboratory of Sichuan Province. Next, 30 g of dried ER was immersed in 6.66 times the volumes (200 mL) of distilled water for 30 min and subsequently extracted at 100°C for 1 h. The extract was further centrifuged at 3,500 rpm for 5 min, after which the supernatant was harvested. Next, the supernatant was in rotary evaporated and lyophilized to obtain ERE, which yielded 4.8 g of dried powder (yield ratio 16%), which was stored at −20°C until further use.

#### 2.3.2 UPLC-MS analysis

The UPLC-MS analysis method has been described previously (Obmann et al., 2011). Briefly, the metabolites in the ERE were identified using a Waters ACQUITY UPLC I-Class (Waters, Milford, MA, United States), a Waters ACQUITY UPLC BEH-C18 column (2.1 × 100 mm, 1.8 μm), and a mass spectrometer (Waters XEVO G2-QTOF). The mobile phase consisted of 0.1% formic acid (FA) in water (A) and 0.1% FA in ACN (B) and was eluted in the following gradient: 0–10 min, 100%–70% (A); 10–25 min, 70%–60% (A); 25–30 min, 60%–50% (A); and 30–40 min, 50%–30% (A). A flow rate of 0.3 mL/min, a column temperature of 40°C and an injection volume of 5 µL were applied. The optimized source voltages and temperatures were set to 2.7 kV and 450°C, respectively, and the mass spectrometer was operated in negative ion mode.

### 2.4 The effect of ER on inflammasome activation

#### 2.4.1 Preparation of medicated serum from ERE

After 5 days of adaptive feeding, 6–8-week-old C57BL/6J male mice were randomly divided into a normal control group and an ERE group (10 mice in each group). The extraction method of ERE is described in section 2.3.1. The ERE dose was calculated according to the “equivalent dose conversion factor between animals and humans,” that is, 0.375, 0.75, or 1.5 g raw ER/kg/day was selected as the dose for mice. The control group was intragastrically administered the same amount of saline (10 mL/kg) once a day for 1 week. Finally, mouse serum was collected and stored at −20°C for *in vitro* experiments.

#### 2.4.2 Cell culture

The method for cell culture was performed as previously described (Wang et al., 2019a; Wang et al., 2020b). Bone marrow cells were obtained from adult C57BL/6J mice and differentiated into murine bone marrow-derived macrophages (BMDMs) supplemented with macrophage colony-stimulating factor (50 ng/mL; MedChemExpress, HY-P7085). BMDMs were incubated in a humidified 5% (v/v) CO_2_ atmosphere at 37°C. BMDMs (1 × 10^6^ cells/well) were seeded in 12-well plates for inflammasomes activation experiments.

#### 2.4.3 Inflammasomes activation

**FIGURE 1:**
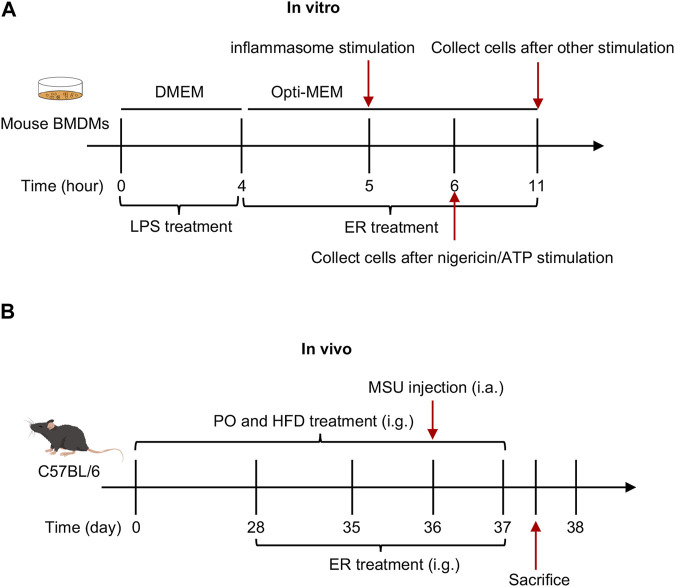
Schematic diagram of *in vivo* and *in vitro* experiments. **(A)** Effect of ERE-medicated serum on the activation of inflammasome *in vitro*. BMDMs were first primed with LPS for 4 h, and then treated with ERE-medicated serum for another 1 h. Finally, various stimuli were used to stimulate for inflammasome activation. **(B)** Effect of ERE on the mouse model of HFD- and PO-induced HUA and MSU-induced gouty arthritis.

Inflammasomes activation was assessed as previously described (Wang et al., 2019b) (Figure 1A).

#### 2.4.4 The activity of caspase-1

To evaluate caspase-1 activity in the supernatants, the Caspase-Glo^®^ 1 Inflammasome Assay was acquired from Promega (G9951). Briefly, according to the manufacturer’s instructions, the Caspase-Glo^®^ 1 reagent was prepared. Caspase-Glo^®^1 reagent was then mixed with the cell supernatants todetermine the luminescence intensity (Tecan, Infinite 200Pro).

#### 2.4.5 ASC oligomerization

The method for ASC oligomerization was performed as previously described (Wang et al., 2019a; Wang et al., 2020b). In brief, the cells were treated with Triton buffer for 30 min. Afterward, 2 mM disuccinimidyl suberate (MedChemExpress, HY-W019543) was added to the resuspended pellets, which were subsequently cross-linked by incubation at 30°C for 30 min. The pellets were resuspended in 1 × SDS loading buffer, boiled for 15 min, and detected by immunoblotting with an anti-ASC antibody.

### 2.5 The effect of ER on the mouse model of HUA and gout

The experimental mice were randomized into a normal control group (vehicle, *n* = 6), an HUA and gout model group (model, *n* = 6), a low dose ER group (ER-L, 0.375 g raw ER/kg body weight, *n* = 6), and a high dose ER group (ER-H, clinical equivalent dose, 0.75 g raw ER/kg body weight, *n* = 6). The extraction method of ERE is described in Section 2.3.1. The mice in the model, ER-L, and ER-H groups were given PO (250 mg/kg by gavage) and fed a HFD (XTHF60, Jiangsu Xietong Pharmaceutical Bio-engineering Co., Ltd.) once daily from Day 0 to Day 37. MSU (2 mg/0.1 mL/mouse) was injected into the ankle joint cavity of the mice in the model group and in the ER group on the 36th day. Mice in the ER group were received low or high doses of ERE once daily from Day 28 to Day 37. The same amount of saline and intragastric gavage (10 mL/kg) were administered once daily to mice in the vehicle and model groups following the same schedule. The diameter of the ankle joints was determined with calipers from 0 to 24 h after MSU injection. At 24 h after MSU injection, blood samples were collected for determination of UA, creatinine (CRE), and blood urea nitrogen (BUN) levels; routine blood examination; and inflammation indices. The mice were subsequently killed with anesthesia. Serum was collected and assayed for mouse IL-1β (Elabscience, E-MSEL-M0003) and mouse TNF-α (Elabscience, E-EL-M3063) in accordance with the manufacturer’s directions. Moreover, kidney samples were collected from each group and stored in for metabolomics, histological analysis and Western blotting (Figure 1B).

### 2.6 Kidney metabolomics analyses

#### 2.6.1 Liquid chromatography (LC) conditions

An ACQUITY UPLC system (Waters, Milford, MA, United States) was used for LC analysis. Chromatography analysis was performed on an ACQUITY UPLC ^®^ HSS T3 (100 × 2.1 mm, 1.8 μm) (Waters, Milford, MA, United States). The column was kept at a temperature of 40°C. The flow rate and injection volume were 0.3 mL/min and 2 μL, respectively. The mobile phases consisted of 0.1% FA in acetonitrile (v/v) (B1) and 0.1% FA in water (v/v) (A1) for LC-ESI (+)-MS analysis. The gradient in Table 1 was used for separation. The analytes were eluted with ACN (B2) and ammonium formate (5 mM) (A2) for LC-ESI (−)-MS analysis. The separation was carried out using the gradient in Table 2.

**TABLE 1:** The separation gradient conditions for LC-ESI (+)-MS analysis.

Time (min)	0.1% formic acid in water (A1) (%)	0.1% formic acid in acetonitrile (B1) (%)
0	92	8
1	92	8
8	2	98
10	2	98
10.1	92	8
12	92	8

**TABLE 2:** The separation gradient conditions for LC-ESI (−)-MS analysis.

Time (min)	Ammonium formate (A2) (%)	Acetonitrile (B2) (%)
0	92	8
1	92	8
8	2	98
10	2	98
10.1	92	8
12	92	8

#### 2.6.2 Mass spectrum conditions

A Q Exactive (Thermo Fisher Scientific, United States) with an ESI ion source was used for mass spectrometric detection of metabolites. MS1 and MS/MS data (full MS-dMS2 mode, data dependent MS/MS) were simultaneously acquired. The parameters used were as follows: envelope gas pressure, 40 arb; aux gas flow, 10 arb; spray voltage, 3.50 kV and −2.50 kV for ESI (+) and ESI (−), respectively; capillary temperature, 325°C; MS1 range, m/z 100–1,000; MS1 resolution, 70,000 FWHM; number of data-dependent scans per cycle, 3; MS/MS resolution, 17,500 FWHM; standardized collision energy, 30 eV; and dynamic exclusion time, auto.

### 2.7 Statistical analyses

The experimental data were presented as the mean ± standard error of the mean (SEM). Significant differences were statistically evaluated using unpaired Student's t test for two groups or one-way ANOVA followed by Dunnett’s *post hoc* test for multiple groups. When *p* < 0.05, the difference was considered to be statistically significant.

## 3 Results

### 3.1 Metabolites isolated from ERE

The chromatogram of ERE was completely isolated within 40 min, as shown in Supplementary Figure S1. MS technique was used to identify the structure of the peaks in the chromatogram. Seven peaks with higher relative contents were selected for analysis. The molecular weight of each peak was determined by MS, and Table 3 shows the details. Peaks 1–7 were identified as dehydroevodiamine, evodine, evodol, evodiamine, rutaecarpine, evocarpine, and dihydroevocarpine, respectively. Next, we determined the relative concentrations in ERE according to peak area, which were 27.47%, 3.15%, 0.9%, 1.71%, 11.07%, 34.14%, and 21.5%, respectively. All of these identified metabolites have a wide range of pharmacological effects, including anti-inflammatory and analgesic effects (Cai et al., 2014; Tian et al., 2019; Li and Wang, 2020), and may be active metabolites through which ER exerts therapeutic effects on HUA and gout.

**TABLE 3:** Identified metabolites in the chromatogram of ERE.

Peak no.	RT (min)	Molecular weight (m/z)	Chemical formula	Proposed structure	Relative concentrations (%)
1	8.67	301.34	C_19_H_15_N_3_O	Dehydroevodiamine	27.47
2	13.66	470.51	C_26_H_30_O_8_	Evodine	3.15
3	14.40	484.5	C_26_H_28_O_9_	Evodol	0.90
4	15.43	303.36	C_19_H_17_N_3_O	Evodiamine	1.71
5	15.77	287.32	C_18_H_13_N_3_O	Rutaecarpine	11.07
6	23.32	339.51	C_23_H_33_NO	Evocarpine	34.14
7	25.54	341.53	C_23_H_35_NO	Dihydroevocarpine	21.56

### 3.2 The ER blocks multiple agonists-induced NLRP3 inflammasome activation in mouse BMDMs

**FIGURE 2:**
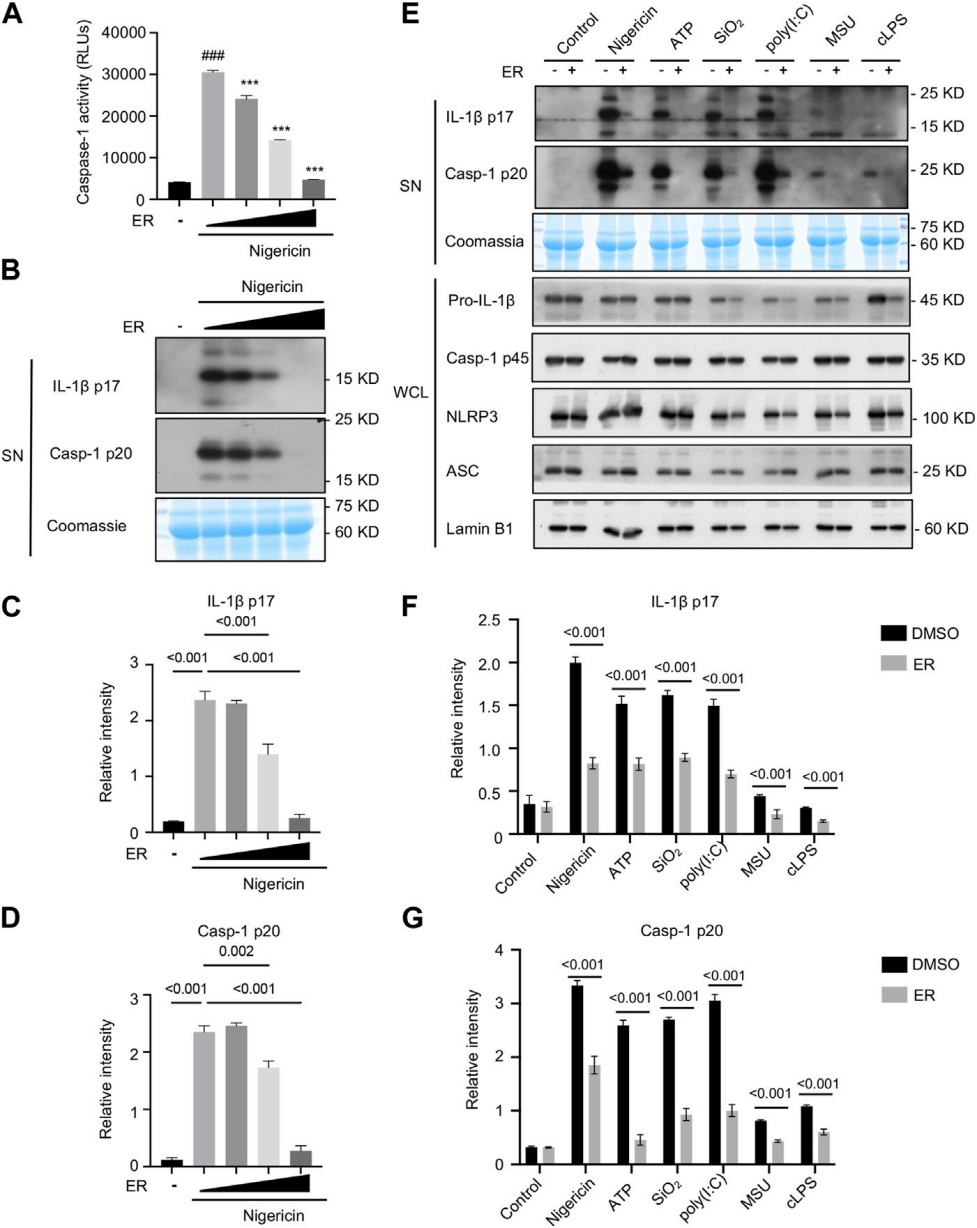
ER inhibits multiple agonist-induced NLRP3 inflammasome activation. **(A)** Recombinant luciferase caspase-1 activity in the supernatant (SN) of LPS-primed BMDMs stimulated with nigericin in the presence or absence of ERE-medicated serum. **(B)** Western blot analysis of cleaved IL-1β p17 and caspase-1 p20 in the SN described in **(A)**. **(C,D)** Relative intensity of IL-1β p17 **(C)** and caspase-1 p20 **(D)** described in **(B)**. **(E)** LPS-primed BMDMs were treated with ERE-medicated serum and then stimulated with nigericin, ATP, poly (I:C), SiO_2_, MSU, or Pam3CSK4-primed BMDMs were treated with ERE-medicated serum and subsequently stimulated with ultrapure LPS. Western blot analysis of cleaved IL-1β p17and active caspase-1 p20 in the SN and pro-IL-1β, caspase-1 p45, NLRP3, and ASC in whole-cell lysates (WCL). **(F,G)** Relative intensity of IL-1β p17 **(F)** and caspase-1 p20 **(G)** described in **(E)**. Coomassie and Lamin B1 served as loading controls in SN **(B,E)** and WCL **(E)**, respectively. The data are expressed as the mean ± SEM from three independent experiments with biological duplicates in **(A,C,D,F,G)**. Significant differences were analyzed using an unpaired Student’s t test in two groups or one-way ANOVA followed by Dunnett’s *post hoc* test for multiple groups: ^###^ *p* < 0.001 vs. the LPS group and ^***^ *p* < 0.001 vs. the LPS + activators group.

LPS-primed BMDMs were exposed to nigericin, an inducer of NLRP3 inflammasome, and then treated with ERE-medicated serum. As shown in Figure 2A, caspase-1 activity in the supernatants was enhanced after nigericin stimulation but was potently inhibited by ERE in a concentration-dependent manner. Furthermore, the results revealed that ERE-medicated serum dose-dependently blocked the cleavage of caspase-1 and IL-1β to their mature forms, caspase-1 p20 and IL-1β p17, respectively (Figures 2B–D). In addition to nigericin, ATP, SiO_2_, MSU, poly (I:C) and intracellular LPS also activate the canonical and noncanonical NLRP3 inflammasome. To further assess the broad-spectrum inhibitory effect of ERE on the activation of NLRP3 inflammasome, BMDMs were stimulated with multiple stimuli before treatment with ERE-medicated serum. Like those of nigericin, IL-1β and caspase-1 cleavage were observed in BMDMs. Interestingly, we observed that ERE-medicated serum markedly reversed these agonists-induced increase in IL-1β production and caspase-1 secretion (Figures 2E–G). These results demonstrateed that ER is a potent and broad-spectrum activator of NLRP3. Evodiamine and rutaecarpine can block NLRP3 inflammasome activation to ameliorate several inflammatory diseases, including gouty arthritis, colitis, and atherosclerosis (Shen et al., 2019; Ding et al., 2020; Luo et al., 2020; Cheng et al., 2023), indicating that ER, which contains these active metabolites, may be able to exert anti-gout effects by inhibiting NLRP3 inflammasome activation.

### 3.3 AIM2 or NLRC4 inflammasome activation is not inhibited by ER

**FIGURE 3:**
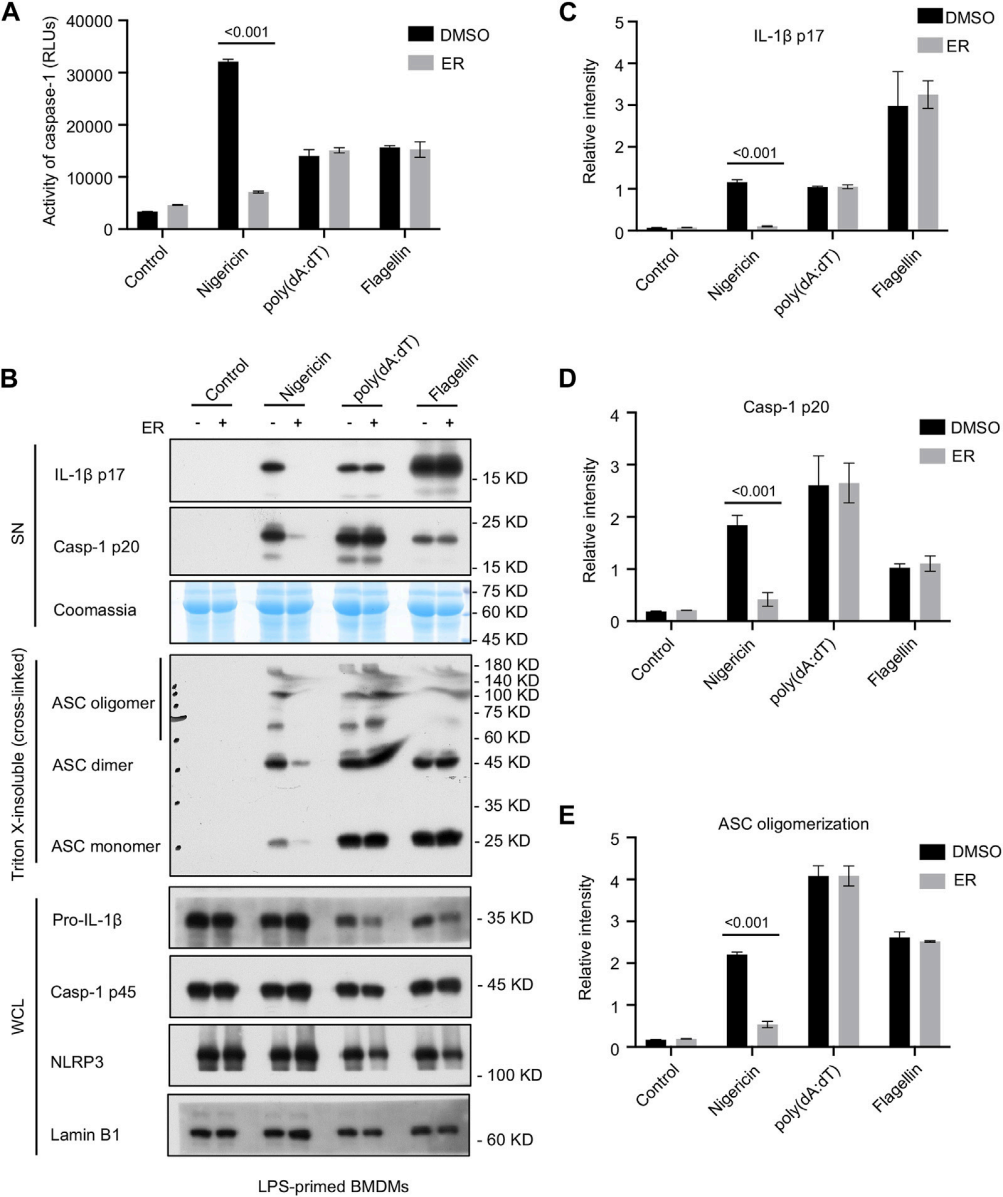
ER suppresses NLRP3-dependent ASC oligomerization, but not AIM2 or NLRC4 inflammasome activation. **(A)** Recombinant luciferase caspase-1 activity in SN from LPS-primed BMDMs stimulated with nigericin, poly (dA:dT) or flagellin, and then treated with ERE-medicated serum. **(B)** Western blot analysis of cleaved IL-1β p17, active caspase-1 p20 in SN, pro-IL-1β, caspase-1 p45, NLRP3, and ASC in the WCL, and ASC oligomerization in Triton X-insoluble (crosslinked). **(C–E)** Relative intensity of IL-1β p17 **(C)**, caspase-1 p20 **(D)**, and ASC oligomerization **(E)** described in **(B)**. Coomassie and Lamin B1 served as loading controls in SN **(B)** and WCL **(B)**, respectively. The data are expressed as the mean ± SEM from three independent experiments with biological duplicates in **(A,C–E)**. Significant differences were analyzed using one-way ANOVA followed by Dunnett’s *post hoc* test for multiple groups: ^***^ *p* < 0.001.

To determine whether the serum concentration of ERE was specific for NLRP3 or more generally, we evaluated the impact of ERE on the activation of AIM2 and NLRC4 inflammasomes. The data showed that ERE did not attenuate caspase-1 cleavage or IL-1β release in response to ultrapure flagellin (Figures 3A–D), suggesting that ERE does not suppress NLRC4 inflammasome activation. The effect of ERE on the AIM2 inflammasome was determined by transfecting BMDMs with the double-stranded cytosolic DNA poly (dA:dT), and the data showed that ERE did not hinder caspase-1 activation or IL-1β release (Figures 3A–D). Taken together, these data support the notion that ERE can selectively suppress the activation of NLRP3 inflammasome.

### 3.4 ER suppresses NLRP3-mediated ASC oligomerization

Given that ASC oligomerization is vital for the recruitment and subsequent activation of caspase-1, we next examined whether ERE mediated NLRP3 inflammasome activation by suppressing ASC oligomerization. Cytosolic fractions from cell lysates were cross-linked, and we noticed an increase in ASC oligomerization after cotreatment with LPS and nigericin in BMDMs, whereas ERE-medicated serum impaired ASC complex formation (Figures 3B, E), verifying that ERE attenuated NLRP3 activation by disrupting ASC oligomerization. These data suggest that ERE may act directly on ASC oligomerization or participate in its upstream processing events to act during assembly.

ASC oligomerization is required for NLRP3 and AIM2 activation but may not be essential for NLRC4 inflammasome activation. In a further study, we tested whether the suppressive effect of ERE on the activation of the NLRP3 inflammasome was mediated by a direct blockade of ASC oligomerization. The data showed that ASC oligomerization was not impaired by ERE-medicated serum under the provocation of NLRC4 and AIM2 stimulators, flagellin and poly (dA:dT), respectively (Figures 3B, E). These results further demonstrated that ERE acts upstream of ASC oligomerization to exert its inhibitory effect. Based on these findings, to further clarify the biological mechanism of ER inhibition of NLRP3 inflammasome, we next will focus on evaluating the effects of the active metabolites in ER on the upstream signaling, including mitochondrial reactive oxygen species, potassium efflux, and lysosomal release, as well as screening target proteins of the active metabolites at a later stage.

### 3.5 ER improves HFD/PO-induced HUA and MSU-induced gouty arthritis

**FIGURE 4:**
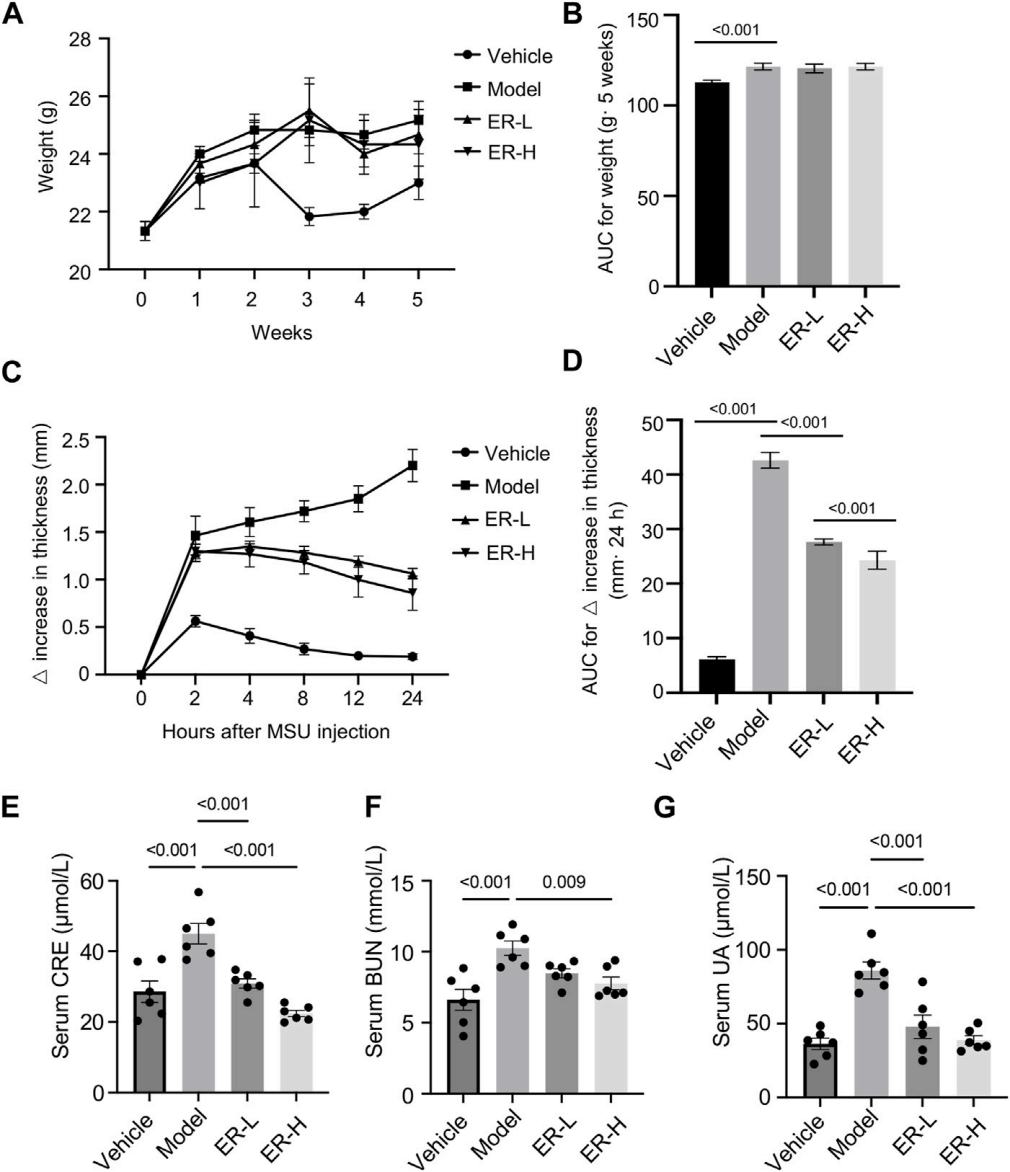
Effects of ER on HFD/PO-induced HUA and MSU-induced gouty arthritis in mice. **(A,B)** The weight of mice. **(C,D)** Joint swelling in the different groups. **(E)** The serum level of Cre. **(F)** The serum level of BUN. **(G)** The serum level of UA. The data are represented as the mean ± SEM (*n* = 6). One-way ANOVA followed by Dunnett’s *post hoc* test was used for statistical significance: ^*^ *p* < 0.05, ^***^ *p* < 0.001.

We first monitored the weight of the mice on a weekly basis during the modeling period. The results showed that the body weights of the mice in the model group were markedly increased in comparison with the vehicle group, while the body weights of the mice in the ER-L and ER-H groups were not significantly greater than those in the model group (Figures 4A, B). Slight changes in the ankle joint thickness ratio were observed in the vehicle group mice, while in the model group, the ankle joint thickness ratio markedly increased after MSU injection, indicating severe ankle joint edema (Figures 4C, D). In addition, within the 24-h observation period, ankle joint edema was somewhat relieved in the mice after ERE treatment. We next evaluated the effect of ERE on liver and kidney function in this model. As shown in Figures 4E–G, the serum UA, creatinine and BUN levels were significantly elevated in the model group compared to those in the vehicle group mice. Treatment with ERE significantly reduced the serum UA, CRE and BUN levels. These results suggest that ER could improve HFD/PO-induced HUA and MSU-induced gouty arthritis with a high safety profile. Moreover, in the current clinical treatment of gout, there are several adverse reactions, such as hepatorenal toxicity and cardiovascular complications, that are associated with most existing agents (Zeng et al., 2023). However, these side effects have not yet been observed in HUA and gout model mice after ER treatment, which deserves further in-depth research and exploration.

### 3.6 ER affects urate transporters in renal tissue

**FIGURE 5:**
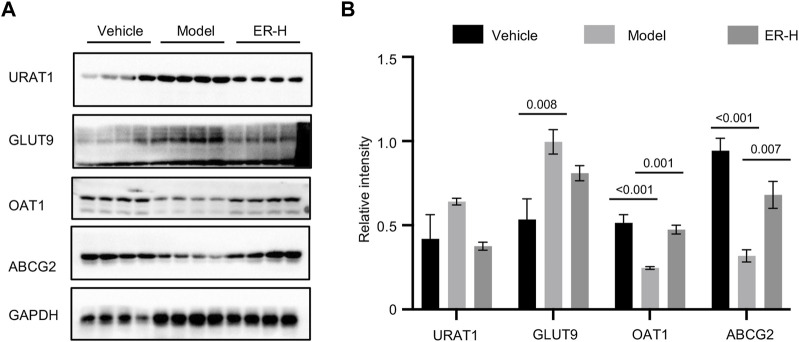
ER reverses the expression of related transporters in renal tissue. **(A)** Western blot analysis of URAT1, GLUT9, OAT1, and ABCG2 in the kidney. **(B)** Relative intensity of URAT1, GLUT9, OAT1, and ABCG2 described in **(A)**. GAPDH was served as a loading control in **(A)**. The data are expressed as the mean ± SEM (*n* = 4). Significant differences were analyzed using one-way ANOVA followed by Dunnett’s *post hoc* test for multiple groups: ^*^ *p* < 0.05, ^***^ *p* < 0.001.

With further research on the pathogenesis of HUA and gout, a series of urate transporter proteins have been identified that play important roles in UA transport *in vivo*. We further evaluated whether ERE affects the levels of urate transporter proteins, such as URAT1, GLUT9, OAT1, and ABCG2, in kidney tissues. The results showed that, compared with those in vehicle group, the expression of URAT1 and GLUT9 was upregulated after HFD/PO treatment. Interestingly, ERE markedly downregulated the expression of these genes compared to the model group (Figures 5A, B). The expression of OAT1 and ABCG2 was clearly downregulated in model group compared to the vehicle group. However, treatment with ERE increased the protein levels of OAT1 and ABCG2 (Figures 5A, B). These results suggest that ER has a remarkable effect on urate transport in HFD/PO-induced HUA and MSU-induced gouty arthritis mouse models. The process of UA excretion and reabsorption from hepatic production to the kidney and intestines involves a variety of urate transport proteins. These results suggest that ER may play a role in lowering UA by promoting the excretion of UA via renal tubules and inhibiting the reabsorption of UA by renal tubules.

### 3.7 ER improves inflammation by suppressing NLRP3 inflammasome activation

**FIGURE 6:**
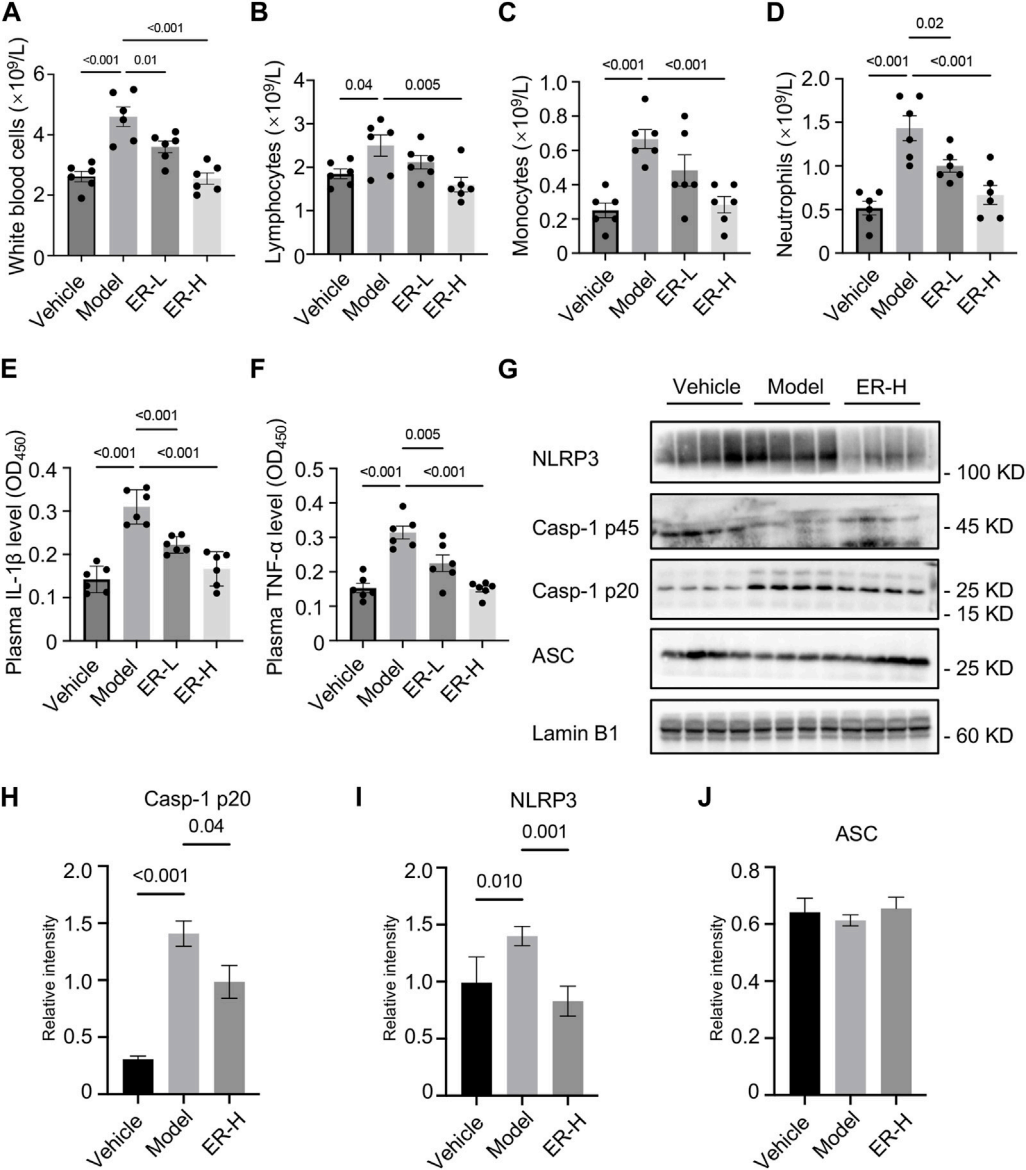
ER improves inflammation by suppressing NLRP3 inflammasome activation in HFD/PO-induced HUA and MSU-induced gouty arthritis mice. **(A–D)** Blood routine tests of white blood cells **(A)**, lymphocytes **(B)**, monocytes **(C)** and neutrophils **(D)**. **(E,F)** Plasma IL-1β **(E)** and TNF-α **(F)** levels in mice. **(G)** Western blot analysis of NLRP3, caspase-1 p45, caspase-1 p20 and ASC in the kidney. **(H–J)** Relative intensity of caspase-1 p20 **(H)**, NLRP3 **(I)** and ASC **(J)** described in **(G)**. Lamin B1 served as a loading control in **(G)**. The data are expressed as the mean ± SEM (*n* = 6 in A-F, and *n* = 4 in **(H–J)**. Significant differences were analyzed using one-way ANOVA followed by Dunnett’s *post hoc* test for multiple groups: ^*^ *p* < 0.05, ^**^ *p* < 0.01, ^***^ *p* < 0.001.

White blood cells, including lymphocytes, neutrophils, monocytes and other types, are important cellular components of the blood and are also an important part of the body’s immune system,. Blood routine tests of the different groups showed that the white blood cell, lymphocyte, monocyte and neutrophil counts in the model group were significantly greater than those in the vehicle group (Figures 6A–D). Importantly, ER treatment reversed the changes in the above cell types (Figures 6A–D), suggesting that ER could change the composition and proportion of blood immune cells, which is of great significance for regulating body immunity. Moreover, in the HUA and gouty arthritis models, IL-1β and TNF-α secretion into the mouse serum was significantly greater than that in the vehicle group. *In vivo* production of IL-1β and TNF-α was eliminated after ER treatment (Figures 6E, F). To validate whether ERE affects NLRP3 inflammasome activation in the experimental model, the expression of inflammasome-related proteins in kidney tissue was analyzed. The results showed that the expression of NLRP3 and active caspase-1 p20 was elevated in HFD/PO-induced HUA and MSU-induced gouty arthritis mouse models (Figures 6G–J). As expected, ER intervention significantly attenuated NLRP3 expression and caspase-1 activation in the kidney tissue (Figures 6G–J). MSU-induced acute inflammation initiation, amplification, and regression, and MSU aggregation leading to chronic inflammation and damage in the joints are the most important core processes in the pathophysiology of gout. The inflammatory response plays a key role in this process. In summary, ER exerts excellent anti-inflammation effects in a mouse model of HFD/PO-induced HUA and MSU-induced gouty arthritis by blocking NLRP3 inflammasome activation.

### 3.8 Kidney metabolomic analysis

#### 3.8.1 Metabolomic analysis method reproducibility assessment

The reliability of the metabolomics profile system is critical for the comparative quantification of spectra between different samples. Thus, to assess the reliability and stability of the metabolomic analysis method, quality control (QC) samples were used. The QC samples tended to cluster and were within the 95% confidence interval according to principal component analysis (PCA) (Supplementary Figure S2A). This is an indication that the instrument is in good working order. In addition, the proportions of features whose variation was ≤30% in the positive ion mode and negative ion mode were 82.8% and 85.8%, respectively (Supplementary Figure S2B). These data show the stability and repeatability of the method used in this study for the analysis of the metabolome.

#### 3.8.2 Multivariate analysis of kidney data

**FIGURE 7:**
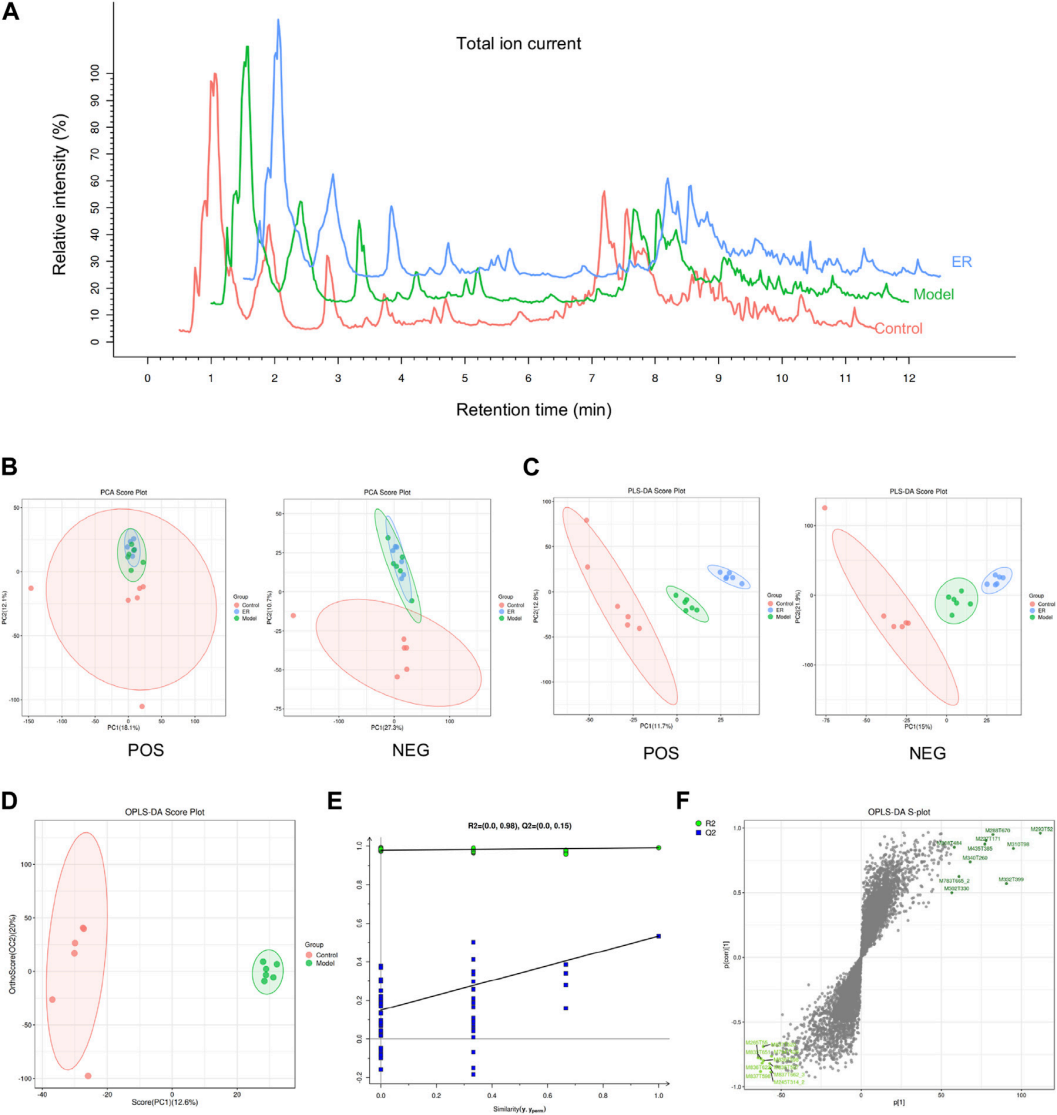
Metabolomic analyses of kidney samples. **(A)** Representative total ion current (TIC) of the vehicle, model, and ER groups in positive ion mode. **(B)** PCA score plots for the vehicle-, model- and ER-treated mice. **(C)** PLS-DA score plots of the three groups based on the kidney metabolic profiles. **(D)** OPLS-DA score plots of the vehicle and model group. **(E)** Validation plots with 100 times permutation tests. **(F)** S-plot of the vehicle and model group from the OPLS-DA model.

All kidney tissues were evaluated by UPLC-MS/MS in positive ion mode to systemically investigate the possible endogenous biomarkers. Under optimal conditions, representative total ion current (TIC) data of kidney samples from the vehicle, model and ER groups were acquired (Figure 7A). A total of 7,710 variables could be identified simultaneously in 12 min. Unsupervised principal component analysis was first applied to detect differences in renal metabolism among vehicle-, model-, and ER-treated mice. As shown in Figure 7B, the renal samples of the three groups were intermingled, and the groups were not able to be effectively distinguished by the plot of the PCA scores. A supervised statistical method for discriminant analysis is partial least squares discrimination analysis (PLS-DA). With this method, a regression model relating metabolite expression to sample class was built to estimate sample class using partial least squares regression. PLS-DA was applied to discriminate the metabolite differences among the three groups, and the PLS-DA score plot revealed that these groups were clearly distinguishable (Figure 7C). The PLS-DA score plots revealed that the model mice were statistically distinguishable from the vehicle- and ER-treated mice, with R^2^Y = 0.998 and Q^2^ = 0.881 and R^2^Y = 0.997 and Q^2^ = 0.919 in the positive and negative ion modes, respectively (Figure 7C). Compared with the unsupervised PCA model, the supervised PLS-DA model was more effective at discriminating the various groups and screening out the differential markers, suggesting that there were some significant differences in the renal endogenous metabolites among the various groups. The model group and the vehicle group could be distinguished from each other, indicating that an HFD in combination with PO-induced HUA model was effectively developed in these mice. The symbols of the ER group were respectively separated from the model group, indicating that ER could regulate the kidney metabolism of HUA.

#### 3.8.3 Identification of potential endogenous biomarkers

To evaluate possible biomarkers related to HUA, an orthogonal projections to latent structures discriminant analysis (OPLS-DA) model was used to determine the difference between the vehicle and model groups. As shown in Figure 7D, the OPLS-DA score plot revealed an outstanding separation between the vehicle and model groups, revealing a significant change in the metabolites of the HUA mice caused by HFD and PO, suggesting that the HUA model had unique metabolic profiles relative to those of the vehicle group. In addition, permutation tests were conducted to verify the OPLS-DA model and to reduce overfitting. The validation of the model with a number of permutations equal to 100 resulted in intercepts of R^2^ = 0.98 and Q^2^ = 0.15 in the mode, indicating that the existing OPLS-DA model has excellent suitability and predictability (Figure 7E). Before being adopted as candidate biomarkers, metabolites were carefully evaluated. These blue dots represent the top 10 contributing metabolites perturbed by HUA, as shown in Figure 7F, S-plot.

**FIGURE 8:**
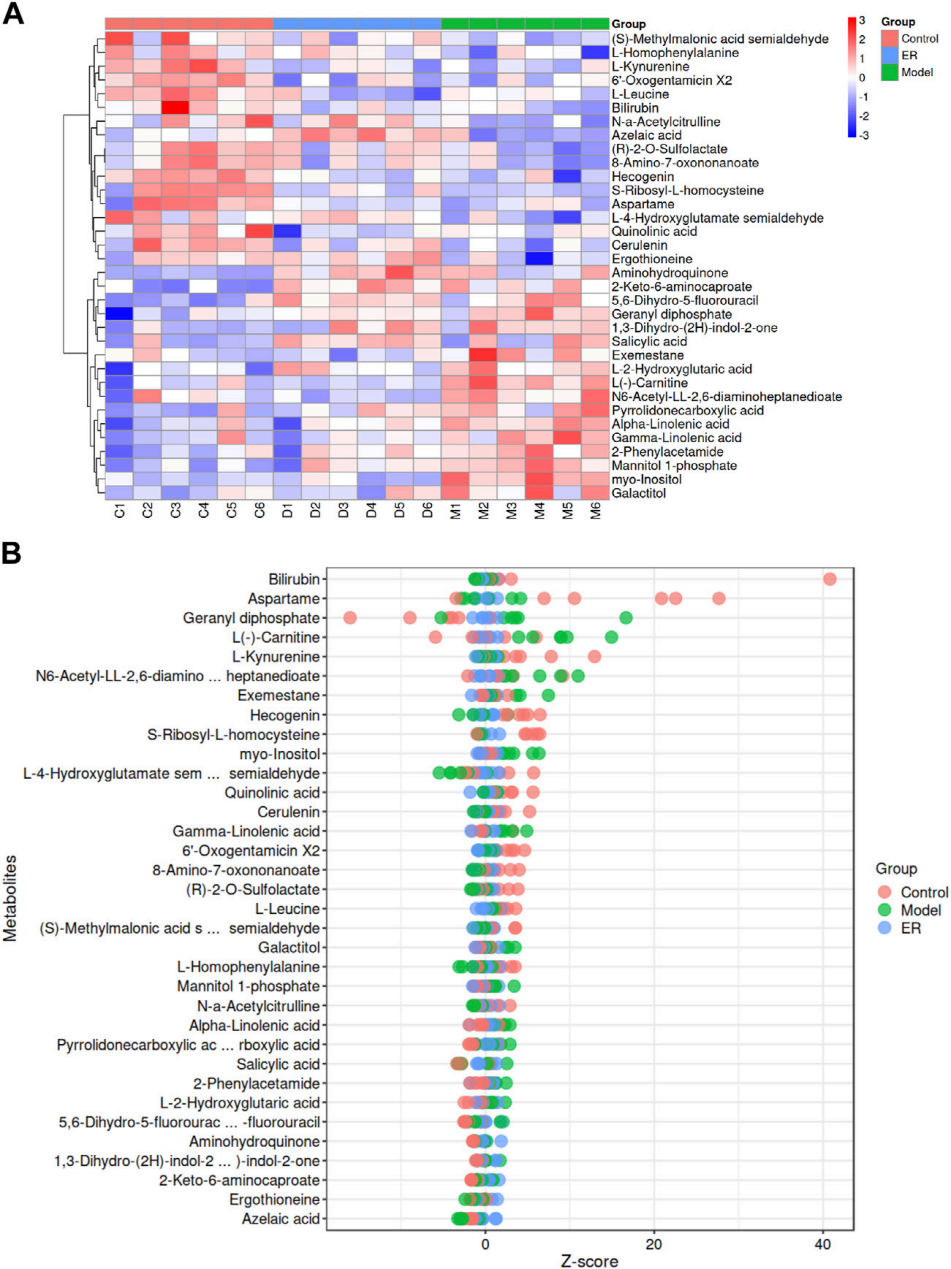
Analyses of the potential endogenous biomarkers in kidney simples. **(A)** Hierarchical clustering heatmap of the 34 differentially abundant metabolites, with the degree of change marked in red (upregulated) and blue (downregulated). **(B)** Z-score map of the 34 differentially abundant metabolites. The closer to the right side, the higher the relative level of the current metabolite in that sample was, and the closer to the left side, the lower the level of the current metabolite was.

The ion properties with a *p*-value< 0.05 and variable importance for projection (VIP) > 1.0 were selected as the differentially abundant metabolites on the basis of the MS/MS results and data from the online database, and the results are shown in Table 4. A total of 34 metabolites were identified as altered in the model group compared to the vehicle group. Seventeen of these metabolites were upregulated, and 17 were downregulated (Figures 8A, B). The levels of aminohydroquinone, pyrrolidonecarboxylic acid, 5,6-dihydro-5-fluorouracil, 1,3-dihydro-(2H)-indol-2-one, salicylic acid, 2-Keto-6-aminocaproate, L (−)-carnitine, myo-inositol, N6-acetyl-LL-2,6-diaminoheptanedioate, exemestane, geranyl diphosphate, 2-phenylacetamide, L-2-hydroxyglutaric acid, galactitol, mannitol 1-phosphate, alpha-linolenic acid and gamma-linolenic acid were increased in the model mice. However, compared with those in the model group, the levels of above 11 metabolites, including pyrrolidonecarboxylic acid, L (−)-carnitine, myo-inositol, N6-acetyl-LL-2,6-diaminoheptanedioate, exemestane, 2-phenylacetamide, L-2-hydroxyglutaric acid, galactitol, mannitol 1-phosphate, alpha-linolenic acid and gamma-linolenic acid, decreased in the ER group.

**TABLE 4:** Identification results and change trends of important differential metabolites.

No.	Metabolites	RT(s)	*m/z*	Formula	P.value	VIP^a^	Control vs*.* model	Model vs*.* ER
1	(S)-Methylmalonic acid semialdehyde	125.7	102.0344	C_4_H_6_O_3_	0.040	1.961	↓	↑
2	Aminohydroquinone	154.8	126.0555	C_6_H_7_NO_2_	0.001	1.708	↑	-
3	Pyrrolidonecarboxylic acid	507.7	130.0505	C_5_H_7_NO_3_	0.031	2.048	↑	↓
4	L-Leucine	251.5	132.1014	C_6_H_13_NO_2_	0.001	1.461	↓	↓
5	5,6-Dihydro-5-fluorouracil	62.4	133.0317	C_4_H_5_FN_2_O_2_	0.003	1.928	↑	-
6	1,3-Dihydro-(2H)-indol-2-one	298.3	134.0602	C_8_H_7_NO	0.033	1.906	↑	-
7	Salicylic acid	329.5	139.1118	C_7_H_6_O_3_	0.013	1.059	↑	↑
8	2-Keto-6-aminocaproate	266.6	146.081	C_6_H_11_NO_3_	0.001	1.983	↑	-
9	L-4-Hydroxyglutamate semialdehyde	109.3	148.0606	C_5_H_9_NO_4_	0.039	1.934	↓	↑
10	L (−)-Carnitine	113.7	162.1126	C_7_H_15_NO_3_	0.000	2.377	↑	↓
11	Quinolinic acid	33	167.0123	C_7_H_5_NO_4_	0.011	1.245	↓	-
12	(R)-2-O-Sulfolactate	144.9	169.9776	C_3_H_6_O_6_S	0.015	2.160	↓	↑
13	L-Homophenylalanine	189.2	180.1025	C_10_H_13_NO_2_	0.043	1.953	↓	↑
14	myo-Inositol	688.7	181.0712	C_6_H_12_O_6_	0.000	2.453	↑	↓
15	8-Amino-7-oxononanoate	216.3	188.1283	C_9_H_17_NO_3_	0.018	2.143	↓	↑
16	L-Kynurenine	394.7	209.1901	C_10_H_12_N_2_O_3_	0.005	2.034	↓	-
17	N-a-Acetylcitrulline	252.6	217.1071	C_8_H_15_N_3_O_4_	0.044	1.797	↓	↑
18	Cerulenin	352.4	223.1153	C_12_H_17_NO_3_	0.026	2.094	↓	↑
19	Ergothioneine	55.8	230.0961	C_9_H_16_N_3_O_2_S	0.035	1.499	↓	↑
20	N6-Acetyl-LL-2,6-diaminoheptanedioate	231.9	233.0915	C_9_H_16_N_2_O_5_	0.017	1.407	↑	↓
21	S-Ribosyl-L-homocysteine	257.6	268.0855	C_9_H_17_NO_6_S	0.003	2.403	↓	-
22	Exemestane	650.4	279.1628	C_20_H_24_O_2_	0.032	1.489	↑	↓
23	Aspartame	186	295.129	C_14_H_18_N_2_O_5_	0.029	1.860	↓	-
24	Geranyl diphosphate	162.6	314.0934	C_10_H_20_O_7_P_2_	0.021	2.072	↑	-
25	Hecogenin	540.6	413.307	C_27_H_42_O_4_	0.001	2.436	↓	-
26	6′-Oxogentamicin X2	438.1	481.2556	C_19_H_36_N_4_O_10_	0.003	1.935	↓	-
27	Bilirubin	659.8	584.4724	C_33_H_36_N_4_O_6_	0.035	1.886	↓	↑
28	2-Phenylacetamide	193.3	134.06	C_8_H_9_NO	0.026	1.969	↑	↓
29	L-2-Hydroxyglutaric acid	99	146.9648	C_5_H_8_O_5_	0.030	1.915	↑	↓
30	Galactitol	332.8	181.0693	C_6_H_14_O_6_	0.048	1.796	↑	↓
31	Azelaic acid	684.7	187.0951	C_9_H_16_O_4_	0.000	1.060	↓	↑
32	Mannitol 1-phosphate	197.2	261.0436	C_6_H_15_O_9_P	0.006	2.240	↑	↓
33	Alpha-Linolenic acid	668.5	277.2171	C_18_H_30_O_2_	0.019	1.998	↑	↓
34	Gamma-Linolenic acid	684.7	277.2169	C_18_H_30_O_2_	0.022	1.785	↑	↓

Coincidentally, the model mice had lower levels of (S)-methylmalonic acid semialdehyde, L-leucine, L-4-hydroxyglutamate semialdehyde, quinolinic acid, (R)-2-O-sulfolactate, L-homophenylalanine, 8-amino-7-oxononanoate, L-kynurenine, N-a-acetylcitrulline, cerulenin, ergothioneine, S-ribosyl-L-homocysteine, aspartame, hecogenin, 6′-oxogentamicin X2, bilirubin and azelaic acid compared with the vehicle group, while the ER group had higher levels of the 10 metabolites than the model group, including (S)-methylmalonic acid semialdehyde, L-4-hydroxyglutamate semialdehyde, (R)-2-O-sulfolactate, L-homophenylalanine, 8-amino-7-oxononanoate, N-a-acetylcitrulline, cerulenin, ergothioneine, bilirubin and azelaic acid.

#### 3.8.4 Differentially abundant metabolite correlation analysis

The aim of the correlative analysis of the different metabolite was to determine the pattern of changes in metabolites and to evaluate the correlation by determing the Pearson correlation coefficient between two of the different metabolites. As shown in Supplementary Figure S3, the vertical and oblique coordinates indicate the names of the differentially abundant metabolites, and the coloring indicates the correlation, where red indicates a positive correlation and blue indicates a negative correlation; the darker the color is, the stronger the correlation. The results showed that (R)-2-O-sulfolactate and 8-amino-7-oxononanoate, alpha-linolenic acid and gamma-linolenic acid, S-ribosyl-L-homocysteine and hecogenin were positively correlated. However, L (−)-carnitine and L-homophenylalanine, myo-inositol and ergothioneine, (S)-methylmalonic acid semialdehyde and geranyl diphosphate, and L-4-hydroxyglutamate semialdehyde and alpha-linolenic acid had strong negative correlations.

## 4 Discussion

HUA and gout have become important diseases that threaten human health, and the exploration of pathogenesis and the selection of therapeutic drugs have been the focus of medical research. The advantage of traditional Chinese medicine in the treatment of gout lies in its overall regulation. Through multiple and multitarget regulatory mechanisms, host UA levels can reach a dynamic equilibrium, which has clinical advantages in accordance with the characteristics of the disease, and the curative effect is precise (Chi et al., 2020; Liang et al., 2021; Wang et al., 2022). This study investigated the anti-HUA and anti-gout effects of the Chinese herbal medicine ER *in vivo* and *in vitro*. In mouse BMDMs, ER can specifically inhibit the activation of caspase-1 and the secretion of IL-1β by inhibiting ASC oligomerization, suggesting its potential as a treatment for HUA and gout. Furthermore, ER significantly blocked the changes in the serum UA, creatinine, and urea nitrogen levels in the PO/HFD-induced HUA mouse model, and also significantly reduced knee swelling in the MSU-induced gouty arthritis model. Moreover, ER also significantly reversed the expression of UA transporters, including ABCG2, GLUT9, URAT1, and OAT1, in renal tissue. These results suggest that ER may significantly improve HUA and gout symptoms. On the other hand, however, the dose used in *in vivo* studies must be of therapeutic relevance and is not meaningful if the starting dose is higher. Therefore, the reasonableness of the dosage selection of ER needs to be further determined based on pharmacokinetic, safety-toxicological, and pharmacodynamic tests.

NLRP3 inflammasome is involved in the pathogenesis of HUA and gout as a sensor of metabolic stress, and targeted inhibition of NLRP3 inflammasome may be a key strategy for the prevention and treatment of HUA and gout (Pellegrini et al., 2019; El-Sayed et al., 2022; Zhan et al., 2022). We further investigated the biological mechanism through which ER protects against HUA and gout. In the HFD- and PO-induced HUA model, ER treatment significantly reduced the number of lymphocytes, neutrophils and monocytes among white blood cells, which play important roles in controlling inflammation levels. The decreases in the serum TNF-α and IL-1β levels also confirmed the potent anti-inflammatory effect of ER. More importantly, the detection of inflammasome-related proteins in kidney tissue showed that ER could significantly suppress the protein expression of NLRP3 and caspase-1 p20 in the mouse model, suggesting that ER exerts a strong anti-inflammatory effect by inhibiting NLRP3 inflammasome activation in HUA mice, thereby ameliorating the symptoms of HUA and gout. The metabolites with relatively high levels in ERE were dehydroevodiamine, evodine, evodol, evodiamine, rutaecarpine, evocarpine, and dihydroevocarpine. Evodiamine and rutaecarpine inhibit several inflammatory diseases, including gouty arthritis, colitis, and atherosclerosis, through suppressing NLRP3 inflammasome activation (Shen et al., 2019; Ding et al., 2020; Luo et al., 2020; Cheng et al., 2023). Dehydroevodiamine has anti-inflammatory effects through the downregulation of proinflammatory cytokines and inflammatory mediators (Wen et al., 2021; Dai et al., 2022; Fu et al., 2022). Experimental studies have shown that the ethanolic extract of ER has better antinociceptive activity, which may be related to its higher contents of evodiamine, rutaecarpine and evodine (Cai et al., 2014). Moreover, the ER biomimetic mixture (mainly including rutaecarpine, evodin, and dehydroevodiamine) has powerful anti-inflammatory activity when applied topically to human skin (Yarosh et al., 2006). The therapeutic effect of Chinese medicine is the result of the combined action of multiple active metabolites in the water decoction. Therefore, when analyzing the anti-inflammatory and analgesic effects of these phytochemicals in ERE together, it is easy to see that the anti-gout effects and anti-HUA effects of ERE are to be expected.

Metabolism is the material basis for maintaining the basic life activities of the body, and normal life activities are based on orderly biochemical reactions. Therefore, we further investigated the effects of ER on various endogenous metabolic activities in HFD/PO-induced HUA models and MSU-induced gouty arthritis models. A comprehensive, systematic and unbiased analysis of all endogenous metabolites in kidney tissues was performed by non-targeted metabolomics to obtain as much metabolite data as possible, which is conducive to the discovery of differentially abundant metabolites or new biomarkers of ER anti-HUA and anti-gout, and plays a positive role in explaining and understanding the nature of the occurrence and development of life activities in the body.

A total of 34 different metabolites were found in the kidney tissues of the mice. Compared with those in the vehicle group, 17 metabolites were upregulated in the model group, while 11 metabolites were downregulated after ER treatment; these included pyrrolidonecarboxylic acid, L (−)-carnitine, myo-inositol, N6-acetyl-LL-2,6-diaminoheptanedioate, exemestane, 2-phenylacetamide, L-2-hydroxyglutaric acid, galactitol, mannitol 1-phosphate, alpha-linolenic acid and gamma-linolenic acid. Compared with the vehicle group, 17 metabolites in the model group were downregulated, while 10 metabolites were upregulated after ER treatment, including (S)-methylmalonic acid semialdehyde, L-4-hydroxyglutamate semialdehyde, (R)-2-O-sulfolactate, L-homophenylalanine, 8-amino-7-oxononanoate, N-a-acetylcitrulline, cerulenin, ergothioneine, bilirubin and azelaic acid. The mechanism through which these differentially abundant metabolites regulate the metabolic function and pathways and subsequently affect the occurrence and development of diseases is the topic of interest.

## 5 Conclusion

In the present work, an NLRP3 inflammasome activation experiment system was used in mouse bone marrow macrophages combined with *in vivo* mouse models to investigate the effect of ER on HUA and gout. Our findings indicated that ER is a potent and specific NLRP3 inhibitor. Importantly, ER exerts potent anti-HUA and anti-gout effects by inhibiting UA production, promoting UA excretion, and improving inflammation, at least in part, through the inhibition of NLRP3 inflammasome activation. Although the UA-lowering and anti-gout effects of ER are well established, the active metabolites that exert the pharmacologic effects and the specific regulatory mechanism merit further investigation.

## Data Availability

The original contributions presented in the study are included in the article/Supplementary Material, further inquiries can be directed to the corresponding authors.
