# Supplementary figures and images for: Anti-inflammatory and uric acid lowering effects of Euodiae fructus on hyperuricemia and gout mice
Source: Front Pharmacol. 2024 Apr 19;15:1296075. doi: 10.3389/fphar.2024.1296075 (PMC11066271; doi:10.3389/fphar.2024.1296075)

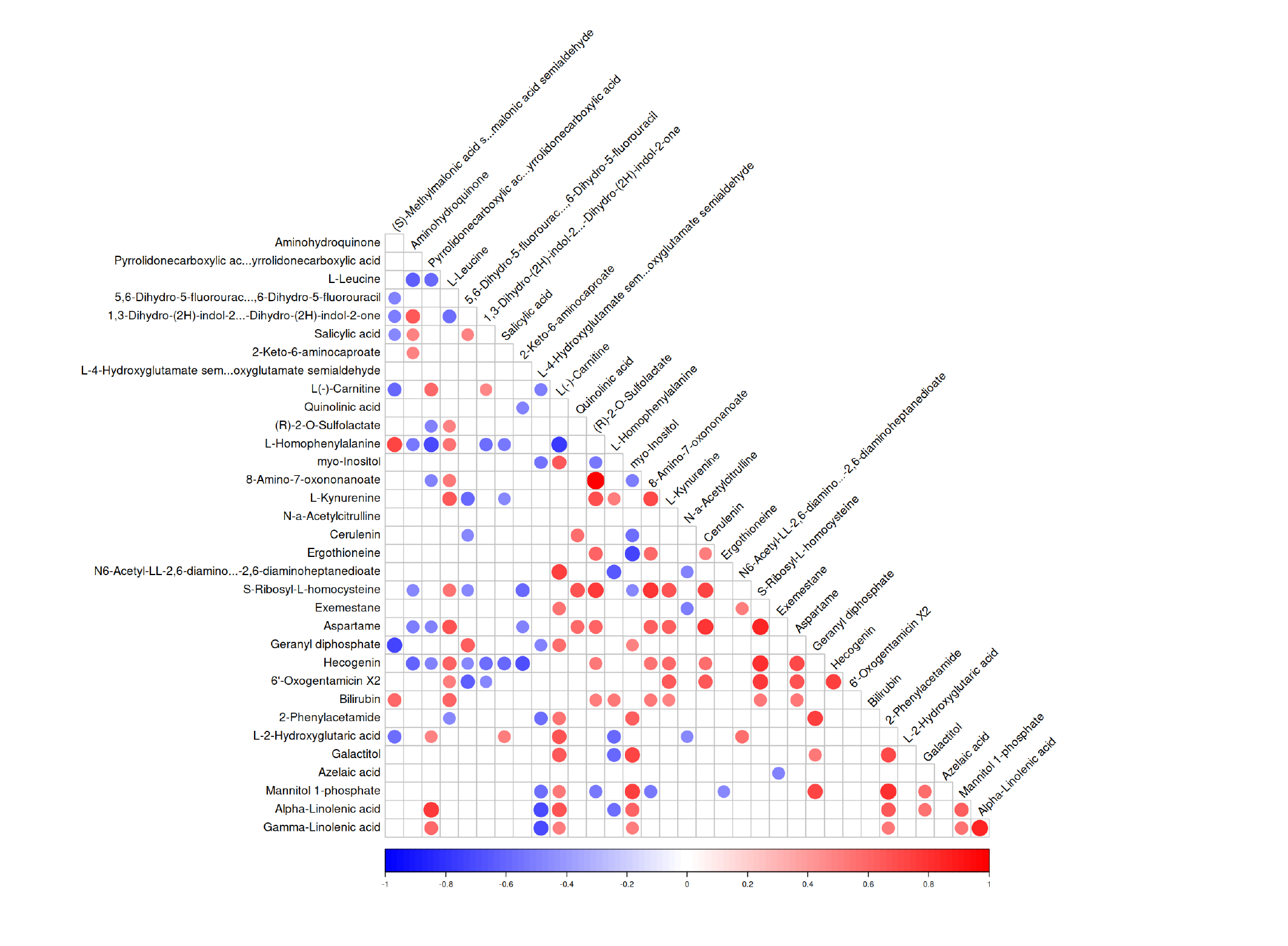

Supplement: Supplementary file 1 [file Image3.TIF]

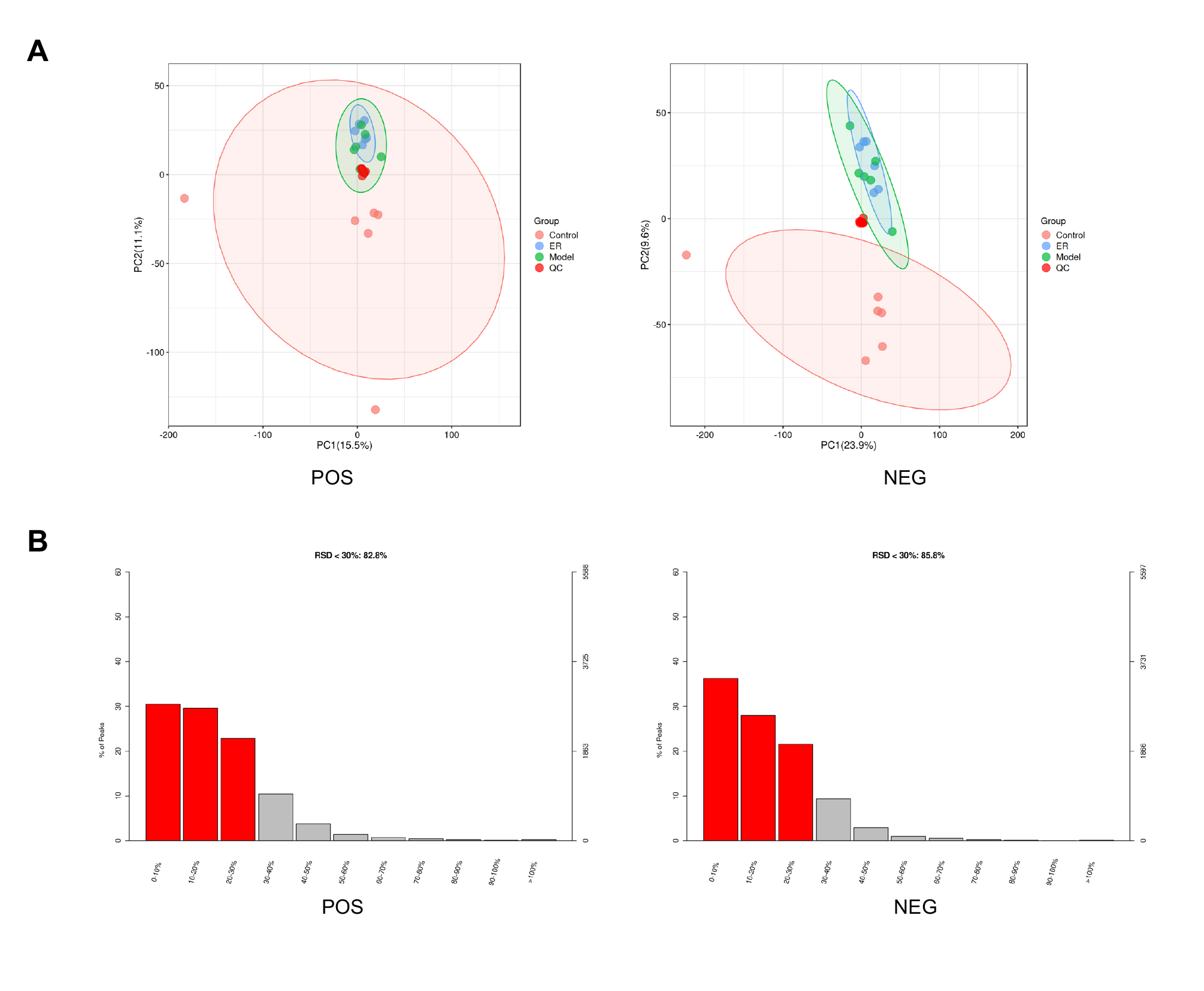

Supplement: Supplementary file 2 [file Image2.TIF]

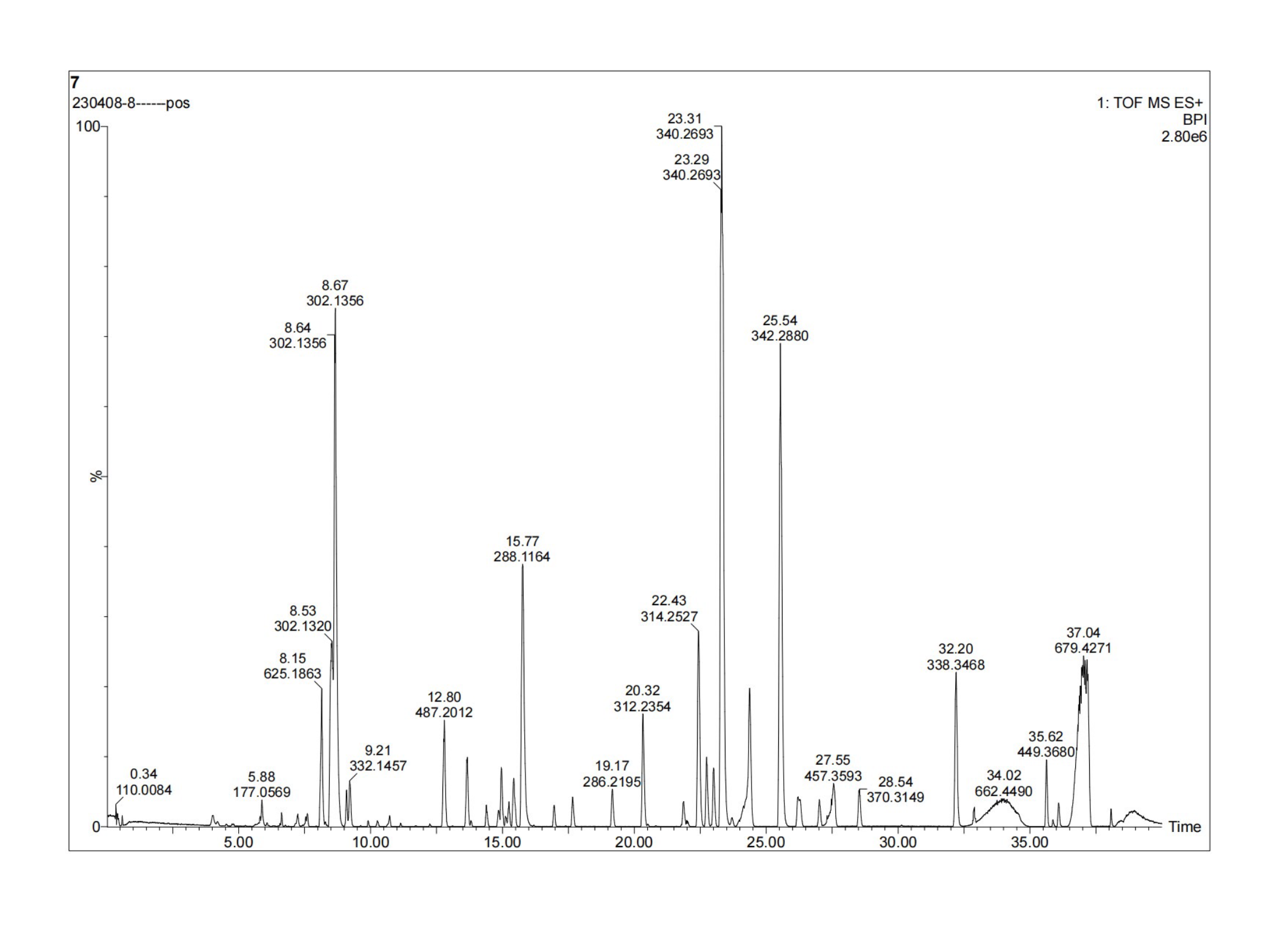

Supplement: Supplementary file 3 [file Image1.TIF]
